# Dual-Valve Culture-Negative Endocarditis: A Case Report

**DOI:** 10.7759/cureus.102659

**Published:** 2026-01-30

**Authors:** Manar Elfatih Mohamed, Yazan Mazen, Chefaa Saleh Shehadeh, Sarah Hussein, Rubina Monga, Muawia Sidahmed Ali Abbas, Leena Abdelrahman

**Affiliations:** 1 Internal Medicine, Dubai Hospital, Dubai Health, Dubai, ARE; 2 Postgraduate Medical Education Division, Mohammed Bin Rashid University of Medicine and Health Sciences, Dubai, ARE; 3 Pathology and Laboratory Medicine/Microbiology, Dubai Health, Dubai, ARE

**Keywords:** bacterial culture-negative endocarditis, dual valve replacement, gastrointestinal symptoms, infective endocarditis, multivalve endocarditis

## Abstract

Infective endocarditis (IE) is a rare but life-threatening condition that poses diagnostic and management challenges, particularly in cases involving atypical presentations or uncommon pathogens. This is a case of a 54-year-old male patient who presented with gastrointestinal (GI) and constitutional symptoms of one week duration, including abdominal pain, intermittent fever, and unintentional weight loss. He tested positive for *Helicobacter* *pylori* gastritis that did not improve with antibiotics and supportive management during admission. Further inpatient evaluation revealed new heart murmurs suggestive of aortic and mitral regurgitation. Due to persistent fever during admission, blood cultures were taken, and an echocardiogram was performed, revealing severe aortic valve endocarditis, anterior mitral valve leaflet perforation, and an aortic root abscess, supportive of IE. Blood culture results were initially positive for *Streptococcus* *paraberis*; however, the rest were negative since then, suggesting that the initial blood culture was a contaminant rather than a true positive culture, and likely attributed to the use of antibiotics prior to obtaining blood cultures. He was diagnosed with blood culture-negative endocarditis and managed by targeted intravenous antibiotics followed by surgical dual valve replacement. Valvular tissue biopsy cultures obtained from dual-valve replacement surgery were negative as well. This case highlights the importance of maintaining a high index of suspicion for IE in patients presenting with generalized symptoms and GI complaints, along with performing systemic physical examinations on all patients, as early recognition can lead to timely diagnosis and improved patient outcomes. Management includes antibiotics and surgical valve replacement if indicated.

## Introduction

Infective endocarditis (IE) is a rare but life-threatening infection of the endocardial surface of the heart that typically involves valves, with an annual incidence of approximately 3-10 per 100,000 individuals [1]. While classic manifestations of IE include fever, fatigue, and heart murmurs, atypical presentations, such as gastrointestinal (GI) symptoms, have been reported. Many of them overlap with constitutional symptoms (e.g., decreased appetite, weight loss, night sweats) [2,3]. This may contribute to delaying diagnosis. Therefore, patients with persistent GI symptoms should be carefully evaluated to rule out rare but serious conditions such as IE.

The diagnosis of IE requires a high index of suspicion, particularly in patients with systemic symptoms, and relies on the Duke criteria, which incorporate clinical, microbiological, and echocardiographic findings [4]. Blood culture-negative endocarditis (BCNE) is a subtype of IE, defined as IE without positive cultures [5]. Causes of BCNE include the use of antibiotics before obtaining blood cultures and pathogens that do not grow on routine blood culture media or have special culture requirements. The former, responsible for partial culture-negative endocarditis, is the most common cause of BCNE, and may occur due to administration of antibiotics prior to obtaining blood cultures in the hospital, or taking antibiotics for another condition prior to IE workup. The latter, responsible for true culture-negative endocarditis, however, may happen without the use of antibiotics before blood cultures are taken, and includes fungi and fastidious organisms: HACEK (*Haemophilus*, *Aggregatibacter*, *Cardiobacterium*,* Eikenella*, and *Kingella*) group and some obligate intracellular bacteria (such as *Coxiella burnetii*, *Bartonella *spp., *Brucella *spp.) [5-7]. Other associated pathogens described in the literature include *Tropheryma whipplei*, and *Mycobacteria*, etc. [7,8].

Non-infectious causes of IE, also called nonbacterial thrombotic endocarditis (NBTE), are less common causes of BCNE, but should be considered. NBTE etiologies include systemic lupus erythematosus (SLE), also known as Libman-Sacks endocarditis, rheumatoid arthritis, and malignancy that has metastasized (called marantic endocarditis) [5,7]. It has been estimated that BCNE accounts for around 2.5-31% of all IE cases [6,8]. Some researchers suggest that antibiotic administration prior to blood culture sampling accounts for 35-74% of BCNE cases [7]. Despite the presence of modified Duke’s criteria for IE, which helps in diagnosing IE in native and prosthetic valves, it has low sensitivity for BCNE [8]. BCNE delays the diagnosis, leading to a worse prognosis and a higher risk of complications like embolic events, heart failure, and abscess formation. The involvement of more than one valve may imply more severe and extensive cardiac lesions [9], which can be seen on echocardiography and are frequently responsible for severe heart failure. Also, the root (periannular abscesses) is the marker of uncontrolled infection, mostly with virulent organisms, leading to severe complications and is associated with high mortality. The aim of this report is to highlight the importance of considering IE in the differentials when patients present with prolonged fever and GI symptoms, and including IE workup (e.g., blood cultures) along with the investigations for the current working diagnosis for the patient.

## Case presentation

A 54-year-old previously healthy man presented to the emergency department with upper abdominal pain for one week. It was associated with nausea, vomiting, nonbloody watery diarrhea, dizziness, generalized fatigue, intermittent fever, decreased appetite, and unintentional weight loss of 10 kg. His symptoms started following a recent trip to Tunisia, which was a week prior to the current encounter. His past medical and surgical histories were not significant.

**Figure 1:**
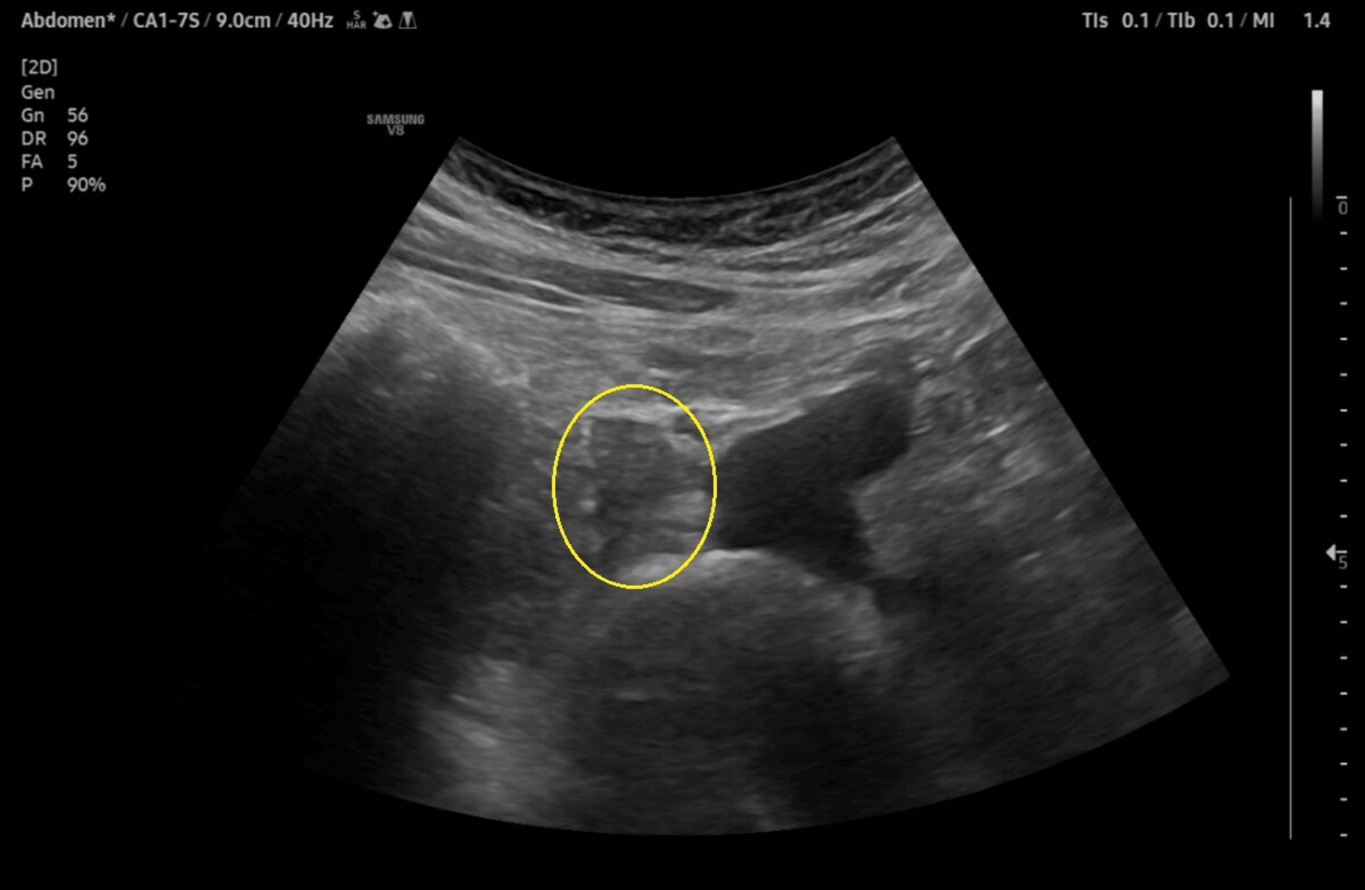
Abdominal ultrasound scan image, showing an irregular shaped soft tissue density lesion (eclipse) in the right lower quadrant, measuring about 16 x 20 mm. Differential diagnosis includes inflammatory changes (panniculitis).

He was afebrile, and vital signs were within normal ranges. Physical examination revealed mild epigastric and right upper quadrant tenderness. There were no mucocutaneous findings, neurological symptoms or signs, or lymphadenopathy. Table 1 shows the initial blood test results obtained in the emergency department; it revealed mild anemia, raised inflammatory markers, and absent leukocytosis. Ultrasound scan of the abdomen showed a small, irregular soft tissue density mesenteric lesion in the right lower quadrant region, shown in Figure 1. The GI panel was negative. However, the *Helicobacter pylori* stool antigen test was positive. He was admitted for acute gastroenteritis and *H. pylori *gastritis for further management and observation. He was started on quadruple therapy: pantoprazole 40 mg twice daily, bismuth subcitrate potassium 140 mg, metronidazole 120 mg, and tetracycline 125 mg, four times daily each for 10 days.

**Table 1:** Blood workup at the time of admission. WBC: white blood cell; GFR: glomerular filtration rate.

Parameters	Patient Values	Reference Ranges
Hemoglobin	11.2	13-17 g/dL
Platelets	242 x10^3	150-410 x10^3 /µL
WBC count	7.1 x10^3	3.6-11.0 x10^3 /µL
Creatinine	0.97	0.70-1.20 mg/dL
Estimated GFR	92.8	>60 ml/min/1.73m^2
Sodium	136	136-145 mmol/L
Potassium	3.7	3.3-4.8 mmol/L
Urea	23	12-40 mg/dL
Bilirubin	0.99	0-1.0 mg/dL
Alanine transaminase	46	0-41 U/L
Aspartate transaminase	26	0-40 U/L
Alkaline phosphatase	108	40-129 U/L
Gamma-Glutamyl transferase	136	8-61 U/L
Albumin	3.4	3.4-4.8 g/dL
Lipase	39	13-60 U/L
C-reactive protein	83.8	<5.0 mg/L
Procalcitonin	0.12	<0.05 ng/ml
Erythrocyte sedimentation rate	39	<11 mm/1hr

**Figure 2:**
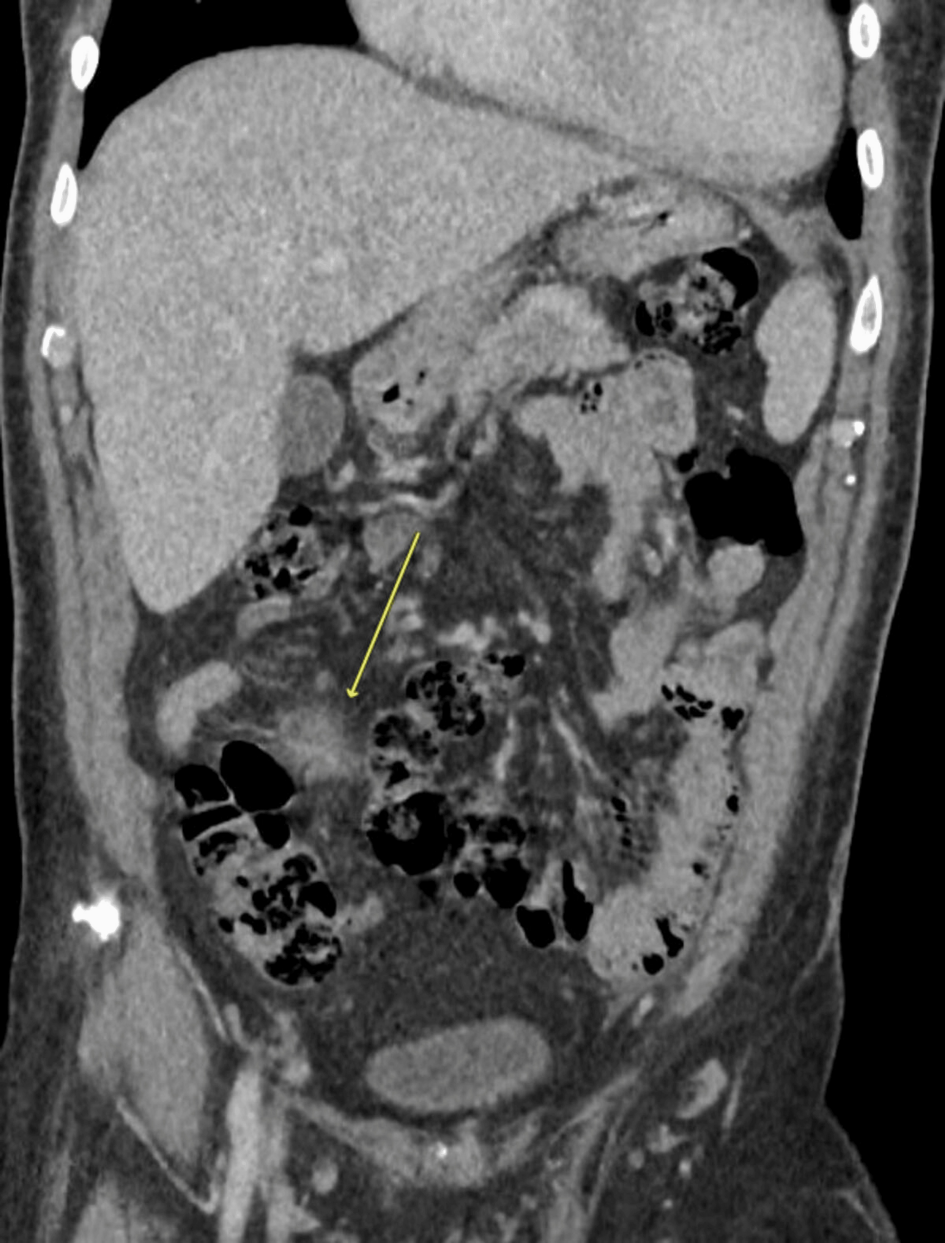
Abdominal CT scan with contrast, coronal view, showing a small soft tissue inflammatory lesion with stranding of the adjacent fat planes (arrow), with the impression of an inflammatory lesion (panniculitis).

During admission, the patient developed a fever. Physical examination was significant for a mild to moderate diastolic murmur at the left lower sternal border and a systolic murmur at the apex that radiated to the left axilla. A computed tomography (CT) scan of the abdomen with contrast showed a small soft tissue density mesenteric lesion with stranding of the adjacent fat planes and local lymph nodes with the impression of an inflammatory lesion, as illustrated in Figure 2.

Blood cultures were obtained, followed by empiric antibiotic therapy of intravenous (IV) piperacillin-tazobactam 4.5 g four times a day, and vancomycin 1 g twice a day. *Streptococcus parauberis *was initially identified in the blood culture using VITEK 2 (bioMérieux SA, Marcy-l'Étoile, France) and matrix-assisted laser desorption/ionization time-of-flight (MALDI-TOF); however, subsequent blood cultures were negative for *S. parauberis *and other pathogens. This may suggest that the initial blood culture was a contaminant rather than a positive culture.

**Figure 3:**
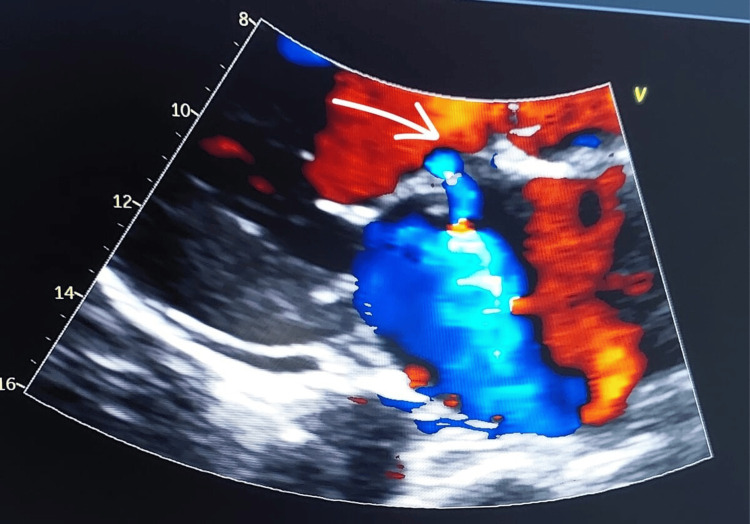
Transthoracic echocardiography (TTE) scan, showing small perforation of anterior mitral valve leaflet (arrow), a complication of infective endocarditis

**Figure 4:**
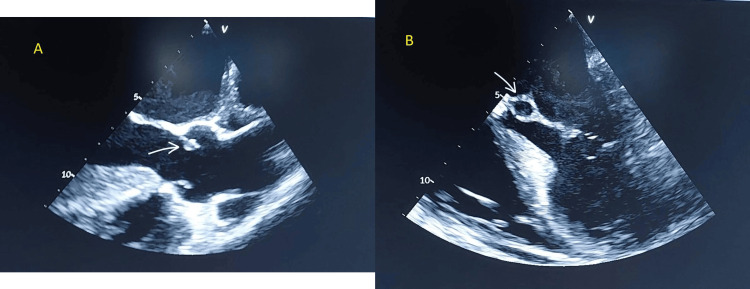
Transesophageal echocardiography (TEE) scan, showing (A) aortic valve vegetation (arrow), and (B) aortic root abscess (arrow), another complication of infective endocarditis and a risk factor for poor prognosis.

Chest X-ray was unremarkable. Transthoracic echocardiography (TTE) showed normal diastolic function and an ejection fraction of 55-60% and evidence of aortic valve endocarditis with mild-moderate aortic regurgitation, and secondary involvement of the anterior mitral valve leaflet, a small perforation due to direct contact with the oscillating aortic valve vegetation (Figure 3). Transesophageal echocardiography (TEE) confirmed the same findings in addition to perforation of the aorto-mitral curtain, moderate mitral regurgitation, aortic vegetations (two vegetations, sizes of 0.8 x 0.8 cm and 1.3 x 0.3 cm), and aortic root abscess (Figure 4). The infectious disease team was consulted, and antibiotics were de-escalated to IV ceftriaxone 2 g once daily, based on *S. parauberis *culture sensitivity.

During admission, the patient had mild abdominal pain despite supportive care; therefore, abdominal CT angiographies were performed, followed by upper and lower endoscopies. The imaging studies revealed patent and normal post-contrast enhancement of the intra-abdominal arterial and venous axis; endoscopy tests were unremarkable.

**Figure 5:**
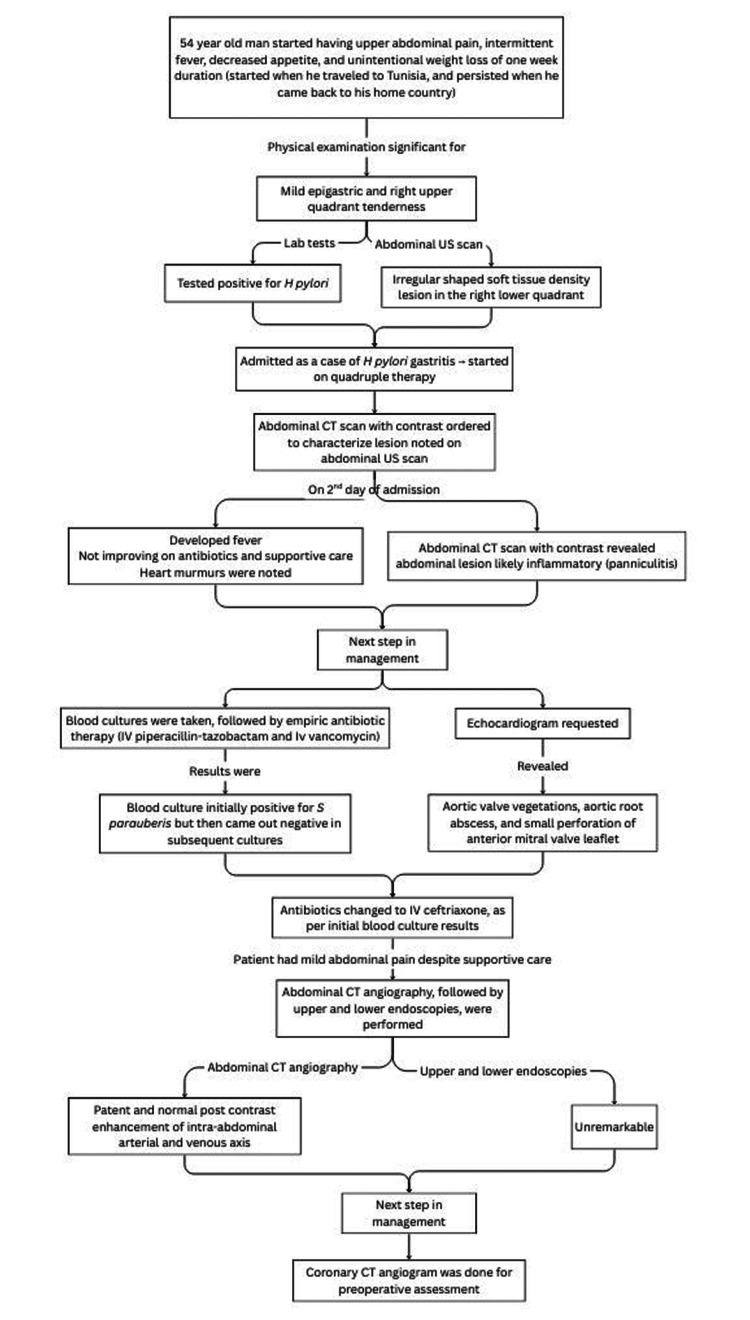
Timeline of the patient’s hospital course. US: ultrasound, CT: computed tomography

The patient was then continued on supportive care, which then resolved. The mesenteric lesion was then determined by a multidisciplinary team (MDT), including radiology, gastroenterology, general surgery, general medicine, and oncology teams, to be an inflammatory lesion (panniculitis), in view of normal abdominal CT angiography and resolution of pain with symptomatic management. It was also determined to be unlikely that it was an embolization (i.e., unlikely related to the valvular vegetations). He subsequently underwent coronary angiography, which was unremarkable as well, and was scheduled for dual valve replacement of the mitral and aortic valves. In the operating room, both valves were seen to be damaged by IE vegetations, were excised, and sent for tissue biopsy cultures. Both yielded negative culture results. Both valves were replaced with mechanical prosthetic valves. He received IV ceftriaxone 2 g once daily for a total of six weeks, made a full recovery, and was discharged on oral warfarin 4 mg daily. Figure 5 shows the timeline of the patient’s illness.

## Discussion

IE is a serious and potentially life-threatening condition [4]. It typically presents with fever, fatigue, weight loss, and heart murmurs [10]. However, it can also present with GI symptoms that mimic gastroenteritis [11] due to several mechanisms, including mesenteric emboli, systemic inflammatory response syndrome, and bacteremia [10]. Several case reports have documented the association of IE with GI-related signs and symptoms: Rezaie Kalantari et al. reported a case of BCNE in a patient who presented with scleral icterus, abdominal pain, vomiting, diarrhea, and fever [12]; Unal et al. described a two-week history of diarrhea and fever in an elderly man, who was later found to have IE [13]. Complications may include embolic events, heart failure, and systemic infection [10].

The clinical overlap between gastroenteritis and IE may delay accurate diagnosis. Duke criteria are used in the diagnosis of IE and consist of major and minor components. Tables 2, 3 demonstrate the definitions and clinical criteria of IE, respectively, as per The 2023 Duke-International Society for Cardiovascular Infectious Diseases Criteria for Infective Endocarditis: Updating the Modified Duke Criteria [14].

**Table 2:** Definitions of infective endocarditis (IE) according to the 2023 Duke-International Society for Cardiovascular Infectious Diseases Criteria for infective endocarditis: updating the modified Duke criteria Data Source: Fowler et al. *The 2023 Duke-International Society for Cardiovascular Infectious Diseases Criteria for Infective Endocarditis: Updating the Modified Duke Criteria*. Clin Infect Dis (2023), 77(4): page 520 [14]; Permission for use was obtained from the licensed content publisher, Oxford University Press.

Clinical Diagnosis Status	Criteria
Definite IE	Pathologic Criteria (one of the following): Microorganisms identified in the context of clinical signs of active endocarditis in a vegetation OR Active endocarditis (may be acute or subacute/chronic) identified in or on a vegetation; Clinical Criteria (one of the following): 2 Major Criteria OR 1 Major Criterion + 3 Minor Criteria OR 5 Minor Criteria
Possible IE	Clinical Criteria (one of the following): 1 Major Criterion + 1 Minor Criterion OR 3 Minor Criteria
Rejected IE	One of the following: Firm alternate diagnosis explaining signs/symptoms OR Lack of recurrence despite antibiotic therapy for less than 4 d OR No pathologic or macroscopic evidence of IE at surgery or autopsy, with antibiotic therapy for less than 4 d OR Does not meet criteria for possible IE, as above

**Table 3:** Clinical criteria for infective endocarditis (IE) according to the 2023 Duke-International Society for Cardiovascular Infectious Diseases Criteria for infective endocarditis: updating the modified Duke criteria * *Staphylococcus aureus*; *Staphylococcus lugdunensis*; *Enterococcus faecalis*; all streptococcal species (except for *Streptococcus pneumoniae* and *Streptococcus pyogenes*), *Granulicatella* and *Abiotrophia* spp., *Gemella* spp., HACEK group microorganisms (*Haemophilus* species, *Aggregatibacter actinomycetemcomitans*, *Cardiobacterium hominis*, *Eikenella corrodens*, and *Kingella kingae*). In the setting of intracardiac prosthetic material, the following additional bacteria should be included as “typical” pathogens: coagulase-negative staphylococci, *Corynebacterium striatum* and *Corynebacterium jeikeium*, *Serratia marcescens*, *Pseudomonas aeruginosa*, *Cutibacterium acnes*, nontuberculous mycobacteria (especially *M. chimaerae*), and *Candida* spp. ** “Blood culture set” is defined as a simultaneously drawn pair of 1 aerobic and 1 anaerobic bottle. “Positive” blood culture set is defined as microbial growth from at least one of the bottles. Blood cultures from separate venipuncture sites are strongly recommended whenever possible for evaluating suspected IE. PCR: polymerase chain reaction; [18F]FDG: 18F-fluorodeoxyglucose Data Source: Fowler et al. *The 2023 Duke-International Society for Cardiovascular Infectious Diseases Criteria for Infective Endocarditis: Updating the Modified Duke Criteria*. Clin Infect Dis (2023), 77(4): page 521 [14]; Permission for use was obtained from the licensed content publisher, Oxford University Press.

Criteria type	Definitions
Major Criteria (includes three separate criterion: microbiologic, imaging, and surgical)	Microbiology (one of the following): Positive blood cultures (either): Microorganisms that commonly cause IE * isolated from two or more separate blood culture sets ** (typical) OR Microorganisms that occasionally or rarely cause IE isolated from three or more separate blood culture sets (nontypical), Positive laboratory tests (either): Positive PCR or other nucleic acid-based technique for *Coxiella burnetii*, *Bartonella* species, or *Tropheryma whipplei* from blood OR *Coxiella burnetii* antiphase I immunoglobulin G (IgG) antibody titer >1:800, or isolated from a single blood culture OR Indirect immunofluorescence assays (IFA) for detection of IgM and IgG antibodies to *Bartonella henselae* or *Bartonella quintana* with immunoglobulin G (IgG) titer ≥1:800
	Imaging (one of the following): Echocardiography and cardiac CT imaging (either) Echocardiography and/or cardiac CT showing vegetation, valvular/leaflet perforation, valvular/leaflet aneurysm, abscess, pseudoaneurysm, or intracardiac fistula OR Significant new valvular regurgitation on echocardiography as compared with previous imaging (worsening or changing of preexisting regurgitation is not sufficient) OR New partial dehiscence of prosthetic valve as compared with previous imaging. [18F]FDG PET/CT imaging. Abnormal metabolic activity involving a native or prosthetic valve, ascending aortic graft (with concomitant evidence of valve involvement), intracardiac device leads, or other prosthetic material
	Surgical: Evidence of IE documented by direct inspection during heart surgery, neither Major Imaging Criteria nor subsequent histologic or microbiologic confirmation
Minor Criteria (includes seven separate criterion: predisposition, fever, vascular phenomena, immunologic phenomena, microbiologic, imaging, and physical examination)	Predisposition (at least one of the following): Previous history of IE Prosthetic valve, Previous valve repair, Congenital heart disease, More than mild regurgitation or stenosis of any etiology, Endovascular intracardiac implantable electronic device (CIED), Hypertrophic obstructive cardiomyopathy, Injection drug use
	Fever - documented temperature greater than 38.0 °C (100.4 °F)
	Vascular Phenomena, confirmed by clinical or imaging evidence of at least one of the following: Arterial emboli, Septic pulmonary infarcts, Cerebral or splenic abscess, Mycotic aneurysm, Intracranial hemorrhage, Conjunctival hemorrhages, Janeway lesions, Purulent purpura
	Immunologic Phenomena (at least one of the following): Positive rheumatoid factor, Osler nodes, Roth spots, Immune complex-mediated glomerulonephritis
	Microbiology - evidence of infection consistent with, but not meeting major criteria (one of the following): Positive blood cultures for a microorganism consistent with IE but not meeting the requirements for Major Criterion OR Positive culture, PCR, or other nucleic acid based test (amplicon or shotgun sequencing, in situ hybridization) for an organism consistent with IE from a sterile body site other than cardiac tissue, cardiac prosthesis, or arterial embolus; or a single finding of a skin bacterium by PCR on a valve or wire without additional clinical or microbiological supporting evidence
	Imaging Criteria: Abnormal metabolic activity as detected by [18F]FDG PET/CT within three months of implantation of prosthetic valve, ascending aortic graft (with concomitant evidence of valve involvement), intracardiac device leads, or other prosthetic material
	Physical Examination Criteria: New valvular regurgitation identified on auscultation if echocardiography is not available (worsening or changing of preexisting murmur not sufficient)

According to Tables 2, 3, the patient in the current case had IE due to the satisfaction of two major criteria: one imaging criterion (presence of valvular vegetations; abscess and leaflet perforation were present as well), and one surgical criterion (direct inspection of IE during heart surgery) [14]. Although the patient was noted to have a heart murmur during admission, it does not necessarily mean that he has developed the new heart murmur acutely. Rather, the possibility of the murmur being missed by the initial physician may be a more likely cause, since heart murmurs can be challenging to note and characterize, including in the emergency department. In addition, the patient presented with signs and symptoms of GI distress or complaint, which may shift a doctor’s attention to GI etiologies and, therefore, investigations related to that system. A similar incident was brought up by Rezaie Kalantari et al., where the patient was reported to have normal heart sounds with no murmurs noted, and later turned out to be an IE [12].

The patient was admitted with suspected acute gastroenteritis and *H. pylori *gastritis and was started on bismuth-based quadruple therapy. Subsequently, he developed fever, prompting the collection of blood cultures and the initiation of broad-spectrum antibiotics. Initial culture identified *S. parauberis*. However, subsequent cultures remained negative. Duke’s major criteria for culture domain states that blood culture should be positive from at least two or three blood culture sets for typical or nontypical organisms, respectively [14]; the patient in our case had only one positive culture, satisfying neither components of the culture criteria. Thus, this may support that *S. parauberis *is a contaminant of the blood culture rather than a true positive test. Cultures from excised valvular tissue were negative, consistent with culture-negative IE.

It is important to obtain blood cultures before starting antibiotics to reduce the risk of BCNE, as well as assist in targeted antibiotic therapy for the pathogens in question. Starting antibiotics early may sterilize the collected blood samples from the associated pathogen. This may have been the case with Rezaie Kalantari et al., where the patient was treated with antibiotics for suspected hepatitis before echocardiography revealed valvular vegetations and shifted the diagnosis to IE [12].

Furthermore, the mesenteric lesion was not determined to be a likely embolic phenomenon as supported by the abdominal CT scans with contrast (which showed a fat stranding pattern) and abdominal CT angiography showing normal findings (i.e., patent and normal vascular post-contrast enhancement). The MDT has therefore supported it as a likely inflammatory lesion. Embolic lesions may present with abscesses or infarctions, as well as mycotic pseudoaneurysms, all of which were not inferred from the imaging studies done [15].

Although patients with multivalvular IE may be thought to have severe presentations (e.g., presenting acutely with signs and symptoms) and hemodynamic instability, there are some case reports that have described patients with multiple valves with hemodynamic stability. For instance, Döngelli et al. described a case of IE involving aortic and mitral valves in a patient who presented with fever, malaise, unintentional weight loss, and night sweats [16]. Some of these constitutional symptoms were also present in our patient. Another case report talked about a middle-aged man who initially presented with fever, shortness of breath, and heaviness in the chest for a duration of five days; he was later found to have an abscess in the mitral-aortic intervalvular fibrosa [17]. Although both patients had bicuspid aortic valves each, the patient in this case does not have a clear past medical or surgical history.

As for the surgical indications, the literature mentions three indications in general: presence of heart failure, uncontrolled infection of IE, and prevention of embolization [6]. The patient did not develop heart failure symptoms because of IE; however, he did have evidence of uncontrolled infection as an aortic abscess (which may be considered as evidence of IE complications to local structures). The patient also had a vegetation size of 1.3 cm, but no signs of severe dysfunction (since he had mild to moderate regurgitation of the valves, not severe). Therefore, the presence of para-valvular abscess and involvement of multiple valves were the indications for surgical management of IE in this patient.

The management of IE includes the use of antibiotics and surgical removal of the affected valves and their replacement with either prosthetic or native valves. Surgical management methods are invasive and associated with various postoperative complications. A previous study reported a 30-day mortality rate of 32% in 25 patients who underwent dual valve replacement with intervalvular fibrous body reconstruction [18]. Patients with bivalvular involvement often need surgery, which consists of radical debridement of all the infected tissue with reconstruction using different types of prostheses; therefore, the surgery may be very complex. The goal should be an early diagnosis of endocarditis to avoid the spread of the infection to more than one valve, to improve the prognosis for those patients [19]. Multivalvular endocarditis is uncommon compared to single-valved endocarditis, with an incidence of 12-30% of all endocarditis cases [20]. Multiple valve endocarditis is a risk factor for poor prognosis [10]; it can also cause severe cardiac damage and heart failure, typically requiring complex surgical intervention with extensive debridement and valve reconstruction [19].

## Conclusions

Early recognition of IE is critical to prevent progression to multiple-valve infection and to improve patient outcomes. This may be enforced by performing systemic physical examination for all patients (even if a patient does not present with typical cardiovascular signs and symptoms, like in this case), and considering taking blood culture from patients with multiple constitutional symptoms before starting antibiotics. An echocardiogram should be performed when a new heart murmur is noted on auscultation, or following blood culture when the suspected index is high.
